# Online CBT Versus Standard CBT for Pediatric Obsessive-Compulsive Disorder

**DOI:** 10.1007/s10578-024-01745-8

**Published:** 2024-08-17

**Authors:** Bernhard Weidle, Lucía Babiano-Espinosa, Norbert Skokauskas, Lidewij H. Wolters, Marit Henriksen, Jostein Arntzen, Anne Skare, Tord Ivarsson, Tricia Groff, Gudmundur Skarphedinsson

**Affiliations:** 1 Regional Centre for Child and Youth Mental Health and Child Welfare, Department of Mental Health, Norwegian University of Science and Technology, Klostergata 46, Trondheim, 7030 Norway; 2 Department of Child and Adolescent Psychiatry, St. Olav’s University Hospital, Trondheim, Norway; 3 Faculty of Behavioural and Social Sciences, Department of Clinical Psychology and Experimental Psychopathology, University of Groningen, Groningen, the Netherlands; 4 Accare Child Study Center, Groningen, the Netherlands; 5 Institute of Neuroscience and Physiology, Sahlgrenska Academy, University of Gothenburg, Gothenburg, Sweden; 6 Dartmouth Health Childrens, Lebanon, NH USA; 7 Faculty of Psychology, University of Iceland, Reykjavik, Iceland

**Keywords:** Obsessive-compulsive disorder (OCD), Cognitive behavioral therapy (CBT), Internet delivered cognitive behavioral therapy, Online CBT, Children, Adolescents

## Abstract

Obsessive-compulsive disorder (OCD), characterized by recurring obsessions and compulsions, affects 1–3% of the childhood population, often leading to severe impairment and reduced quality of life. Cognitive behavioral therapy (CBT) is well-documented as first choice treatment for pediatric OCD. Traditionally delivered face-to-face CBT has limitations in terms of accessibility, availability, and quality of delivery. Online CBT using video conferencing (online-CBT) at home aims to address some of these barriers. In this pilot study, we aimed to compare acceptability, feasibility and effectiveness of online CBT against face-to-face CBT. Online CBT outcomes of 29 children with OCD were analyzed benchmarked against outcomes of face-to-face CBT (*n* = 269) from the Nordic Long-term OCD Treatment Study, the largest CBT follow up study in pediatric OCD to date. Acceptability rated by online CBT participants and their parents was very high (Client Satisfaction Questionnaire total scores about 30, range 8–32). Feasibility assessed as dropout rate was comparable to NordLOTS (10.3% versus 9.7%). The online CBT group compared to NordLOTS showed a higher response rate (90% versus 60%; *p* = .002) and remission rate (81% versus 53%; *p* = .231). Our results suggest that the trusting therapeutic relationship necessary for demanding exposure-based treatment can be established by online CBT. Online CBT seems to be at least as effective in reducing OCD symptoms than standard CBT. Trial ID: ISRCTN37530113.

## Background

Obsessive-compulsive disorder (OCD), defined by the presence of recurrent obsessions and/or compulsions affects about 1–3% of children and adolescents [16, 23]. Without treatment, the disorder often leads to functional impairment [36, 43] and reduced quality of life [45]. There is a strong evidence base for cognitive behavioral therapy (CBT) with exposure and response prevention (ERP) as the first choice treatment for pediatric OCD [19, 31, 34, 48]. However, various organizational, geographic and practical barriers to treatment have been identified that limit the availability and accessibility of CBT [29, 46]. In a recent review, Farrell et al. [13] described these barriers to access evidence-based treatment for youth with OCD as an unacceptable “treatment and quality gap”. The treatment gap is characterized by the large number of children with OCD worldwide who never gain access to treatment. Meanwhile, the quality gap refers to those who do access services but do not receive evidence-based CBT/ERP. The authors exemplified the treatment gap with “the difference between the 1 in 50 children who suffer from OCD at any point in time, and the negligible proportion who ever receive evidence-based treatment”. Another example for the treatment gap is frequent delays in access to care. Dell’Osso described a duration of untreated illness of around 7 years for OCD in the majority of the reports worldwide [10]. In a study surveying 19 expert centers from 15 countries on treatment praxis for OCD, ERP was received by only 31.5% of the 4086 participants for whom this information had been recorded. Most centers reported that ERP was usually available in teaching hospitals or specialized clinics, but that access to adequately trained ERP therapists was generally difficult [7]. An example for the quality gap is given in a study by Krebs et al. [26]. The authors evaluated treatment resistance in young people with severe OCD and non-response to previous CBT and selective serotonin reuptake inhibitors referred to a specialist clinic. They also assessed quality of previous CBT in a sub-sample, concluding that previously delivered CBT was inadequate in 95.5% of cases. The most common inadequacy was insufficient focus on exposure techniques.

As a potential solution for these gaps, Farrell et al. [13] propose a stepped care model including the use of new technologies and alternative models for delivering CBT. Internet-delivered CBT, often denominated as iCBT or online CBT, can refer to entirely remotely therapist-delivered CBT, or it can refer to therapist-assisted self-help programs with variable intensity of therapist involvement. All these interventions have the potential of being an important tool for addressing both the treatment and quality gaps. Despite an extensive growth of internet-based treatment strategies for various mental disorders in children and adolescents, research in online CBT for pediatric OCD is limited. Two recent systematic reviews identified only six and eight eligible studies, respectively, about online CBT for pediatric OCD [6, 35]). Both reviews found promising results, but still a very limited evidence base warranting more research.

Prior to COVID-19 pandemic, we developed an innovative treatment package eCBT (enhanced CBT) for children and adolescents with OCD consisting of a combination of videoconferencing sessions, and face-to-face sessions, and a smartphone application with psychoeducation videos, and online ratings allowing for direct feedback to the patient. The eCBT package has been described in detail elsewhere [47]. Feasibility, acceptability and effectiveness evaluation of this package suggest that eCBT is highly accepted and feasible with high scores on the Client Satisfaction Questionnaire (range 8–32, mean score 27.6 -SD 0.67 for children and 29.5-SD 3.74 for parents) and no patients dropping out of treatment [5]. In a pilot open trial benchmarked against outcomes of traditional face-to face CBT from the Nordic Long-term OCD Treatment Study (NordLOTS), the largest pediatric OCD CBT study to date, eCBT has shown non-inferiority [4].

When the COVID-19 pandemic lockdown measures were introduced in Norway in March 2020, we decided to offer a modified eCBT package, where all face-to-face-sessions were replaced by online sessions, (i.e., 100% remote treatment). Since we had established videoconferencing tools during the eCBT project with combined treatment, we were able to offer 100% online treatment from the third day of the lockdown.

Our initial hypothesis was that establishing rapport, as well as motivating and engaging young people in the treatment process, would be more challenging without direct face-to-face sessions, potentially leading to lower acceptance, a higher dropout rate, and less favorable outcomes.

Therefore, we wished to test this hypothesis with the same tools we had used in the evaluation of the combined eCBT treatment. The aim of the present study was to evaluate the acceptability, feasibility, and effectiveness of this online treatment package, where the core elements of face-to-face CBT were delivered 100% online. For this reason, we conducted a pilot open trial to compare online CBT outcomes benchmarked against outcomes from NordLOTS CBT. The NordLOTS established effectiveness and feasibility of CBT for OCD in the context of the Scandinavian healthcare system, including both specialized OCD clinics and general child psychiatric outpatient clinics [18, 32]. Since online CBT was originally developed based on NordLOTS standards, and both assessment and sample characteristics were similar, we could use NordLOTS outcome data as a reference frame for comparison with the online CBT modification.

## Methods

### Design

This pilot study evaluated outcomes for 29 patients treated with 100% online CBT for OCD from February 2020 to November 2021. Participants were assessed at pre- and post-treatment, and at 3-, 6- and 12-month follow-up. At follow-up assessment points, participants were encouraged to continue their good work, but no additional sessions scheduled nor any medication for OCD added.

Acceptability measures were applied once at post-treatment. Dropout rate and a questionnaire to rate barriers to treatment participation were used to evaluate feasibility. Efficacy was benchmarked against measures from face-to-face delivered CBT from the NordLOTS.

### Participants

Participants were aged 7–17 years; had a primary DSM-5 diagnosis of OCD; and a severity score of ≥ 16 at the Children’s Yale-Brown Obsessive-Compulsive Scale (CY-BOCS). Exclusion criteria were having a psychiatric comorbidity with higher treatment priority making participation clinically inappropriate (e.g., primary anorexia nervosa, depression with suicidality or psychosis), intellectual disability, ongoing other psychological treatment for OCD or insufficient understanding of the Norwegian language. Concurrent medications for non-OCD disorders were allowed during the study. Exclusion criteria were identical with the NordLOTS study, with the exception that patients with OCD and comorbid autism spectrum disorder (ASD) were not excluded from the online CBT study.

### Online CBT

The online CBT study was conducted in child and adolescent mental health services (CAMHS), St. Olav’s University Hospital, Trondheim, Norway (*n* = 29). Between February 2020 and October 2021, a total of 74 children and adolescents with suspected OCD were routinely referred to CAMHS, St. Olav’s University Hospital, Trondheim. All of them were evaluated for participation in the study. There was no specific recruitment for the study. Of these, 36 did not fulfill inclusion criteria. There was a variety of reasons for exclusion of these 36 referrals. Most frequently (*n* = 21) the evaluation concluded with another disorder than OCD (ASD, tic disorder, non-OCD anxiety disorder, ADHD, intellectual disability, misophonia). In six cases, the children did not wish treatment and did not give their consent, two of these had a diagnosis of comorbid ASD and another one had suspected, but not diagnosed ASD at the timepoint for referral. Three children had subclinical OCD with CYBOCS scores < 15. Three children were under 7 years of age. Two referrals were postponed due to incomplete concomitant ADHD assessment which was delayed due to COVID measures. Finally, one referral moved to a different part of the country after assessment.

**Fig. 1:**
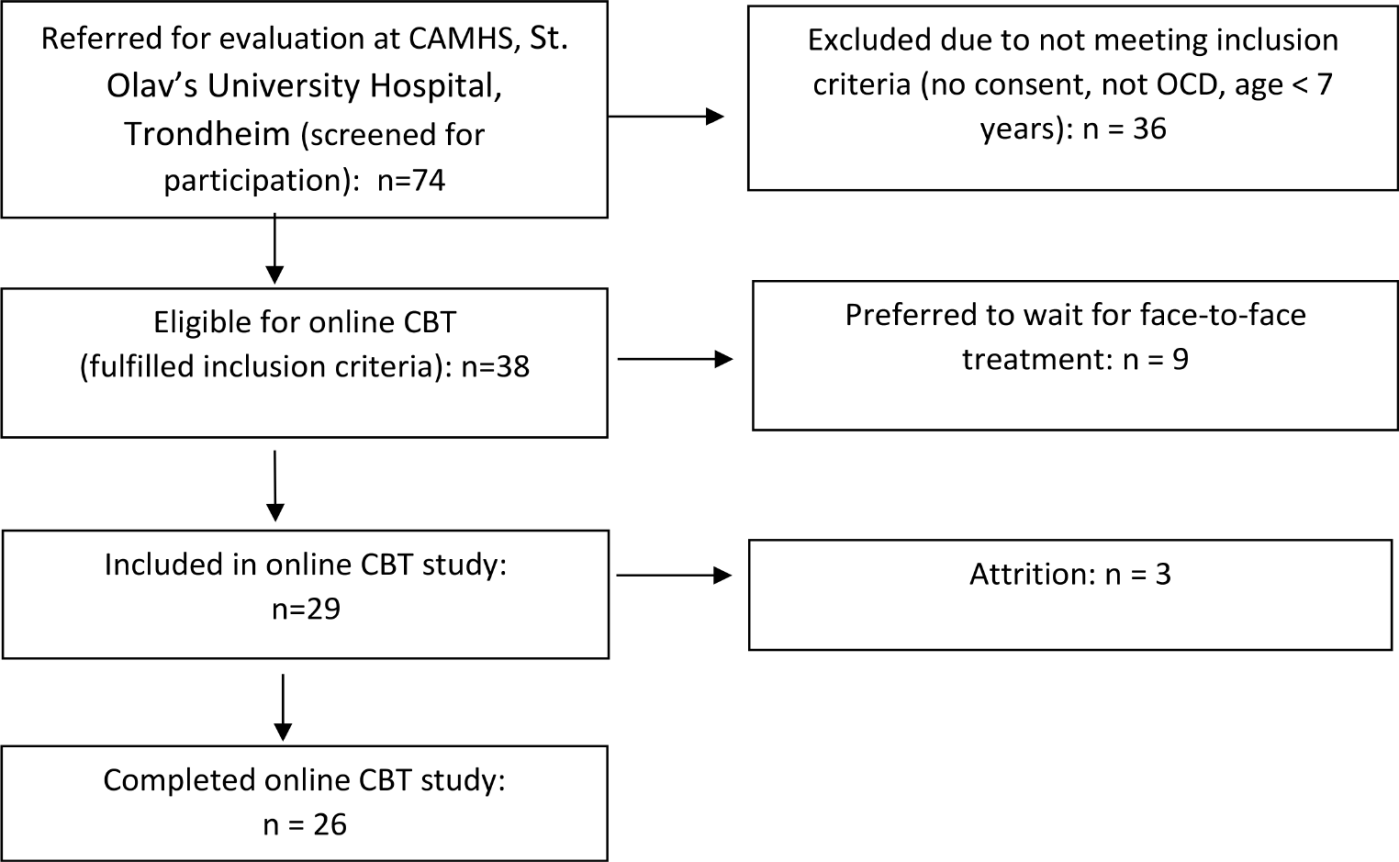
Patient recruitment flowchart

The 38 eligible patients were informed about the study and 29 agreed to participate (76%), while nine (24%) preferred to wait until combined face-to-face and web-based treatment would be available again after the lockdown (see patient recruitment flowchart, Fig. 1). As a result, the final sample consisted of 29 participants.

For an overview of participants and outcomes see Table 1. Mean age of participants (*N* = 29) at baseline was 12.7 years (SD 2.6), with more female (62%) than male participants, and 96.5% having a Scandinavian background.

**Table 1:** Comorbidities, number of sessions and CY-BOCS total scores at baseline, post-treatment, and at 3-, 6- and 12-month follow-up (*n* = 29)

Number	Comorbidities (K-SADS)	Number of sessions	CY-BOCS total score				
			Pre	Post	3 m	6 m	12 m
1	ADHD	22	30	15	11	5	0
2		15	32	9	NA	5	0
3		12	25	0	0	0	0
4		8	21	0	0	0	4
5	Anxiety disorder	7	21	0	0	0	0
6	Tourette’s syndrome	10	23	8	3	3	0
7		11	26	2	0	0	0
8	Anxiety disorder	7	26	0	0	0	0
9	Anxiety disorder	10	21	3	8	13	12
10		12	21	0	6	9	11
11	Anxiety disorder, Tourette’s syndrome, ASD	18	30	8	7	0	0
12	ADHD	11	22	14	14	10	0
13	Transient tic disorder	12	32	2	16	9	0
14		12	25	2	0	0	0
15	ADHD, Transient tic disorder	8	25	0	0	0	0
16	Transient tic disorder	14	22	7	0	0	0
17	Depressive disorder	7	21	0	0	0	0
18	Adjustment disorder (mixed anxiety and depressive)	18	26	10	7	4	6
19	ADHD, Tourette’s syndrome	16	30	17	10	9	4
20	ASD	17	19	10	8	5	6
21	Anxiety disorder	15	28	0	8	18	8
22		7	22	2	2	NA	0
23		13	36	0	0	0	0
24	ADHD	10	24	6	15	6	3
25		9	28	7	0	10	7
26	ASD, Tourette’s syndrome	22	35	24	NA	NA	NA
27	ASD, ADHD	Dropout					
28	PTSD at baseline, later ADHD, ASD	Dropout					
29	None at baseline, later ASD	Dropout					

K-SADS – Kiddie Schedule for Affective Disorders and Schizophrenia; CY-BOCS – Children’s Yale-Brown Obsessive-Compulsive Scale; ADHD – Attention Deficit Hyperactivity Disorder; ASD – Autism Spectrum Disorder; PTSD – Post-Traumatic Stress Disorder; NA – Not available

### NordLOTS

A total of 269 children and adolescents, recruited at five main study sites in Denmark, Sweden, and Norway between September 2008 and June 2012, were included in the NordLOTS. Assessment and treatment were performed in 18 public community mental health clinics and at two specialized OCD clinics [42]. Mean age of the NordLOTS participants was 12.8 years (SD 2.7) and 51.3% were female. Ethnicity was also primarily Scandinavian (97%). In the NordLOTS 241 (89.6%) of the 269 participants completed the treatment.

### Treatment

#### NordLOTS

NordLOTS treatment consisted of 14 sessions of weekly individual manualized CBT with ERP, based on the study protocol of March et al. [28], modified by adding more extensive family participation [37] and adapted to fit Nordic culture [44]. Treatment included psychoeducation about OCD and a treatment model of gradual exposure to anxiety provoking situations. Information about the NordLOTS treatment is given in details elsewhere [20, 41, 42].

#### Online CBT

The basic content of online CBT was comparable with the NordLOTS treatment. Like the NordLOTS, online CBT contained psychoeducation, ERP, cognitive interventions, and relapse prevention, with the focus on ERP. An app system, including psychoeducation videos and online ratings with direct feedback to the patient, was used to support the treatment structure. The distribution of treatment sessions over time was 15 sessions, similar to the NordLOTS. However, online sessions were in general shorter than the face-to-face sessions scheduled for 90 min in NordLOTS. This difference was compensated for by offering additional shorter online sessions to maximum of 22 sessions if needed, which was the case for six patients. Total use of therapist time was comparable to NordLOTS. Mean number of treatment sessions per patient was 12 (Table 1). In online CBT and in NordLOTS, parents were involved by default.

### Measures

#### Acceptability Measures

The *Client Satisfaction Questionnaire 8 (CSQ-8)* is an eight items questionnaire assessing client satisfaction with mental health services. Each item is rated on a 4-point Likert scale, yielding total scores between 8 and 32, with higher scores signifying a higher level of acceptability [3, 9]. The CSQ-8 was used to examine treatment satisfaction of both participants and parents.

#### Feasibility Measures

Primarily, we used treatment drop-out to measure feasibility. In addition, the therapists completed a session integrity form after each session and the forms were inspected for deviations.

The modified *Barriers to Treatment Participation Scale (BTPS)* is a questionnaire that measures perceived barriers to participate in treatment [22]. The original form includes obstacles related to participation, perceptions that treatment is (too) demanding, not helpful, or of little relevance to the child’s problems, and a poor alliance with the therapist. We modified the original form by adapting questions to be relevant for our study. The adapted BTPS consisted of two versions: 27 items for the parents’ version and 15 items for the childrens’ version, which are scored on a 5-point Likert scale, with 1 indicating “never a problem” and 5 “very often a problem”.

#### Efficacy Measures

The *Children’s Yale-Brown Obsessive-Compulsive Scale (CY-BOCS) * [38] is a clinician-rated semi-structured interview for the assessment of the severity of OCD symptoms in children and adolescents. Psychometric properties have high internal consistency (ɲ = 0.90) and test-retest stability for the total score (ICC = 0.79). The validity has good inter-rater agreement (ICC 0.84 for the total score) [14], 40]. The instrument has two subscales, one for obsessions and one for compulsions. The CY-BOCS total score (range 0–40) is the sum of the subscale scores (range 0–20).

*The Clinical Global Impression (CGI)* measures symptom severity, treatment response, and efficacy in treatment studies. Two scales are available, one for severity and one for improvement. The Severity Scale (CGI-S) is a 7-point scale from 1 (normal) to 7 (extremely ill). The Improvement Scale (CGI-I) is also a 7-point scale from 1 (very much improved) to 7 (very much worse) [8]. The CGI is included in the CY-BOCS. The CY-BOCS and CGI were applied at all observation points (baseline, post treatment and follow up 3, 6 and 12 months).

#### Other Measures

The *Schedule for Affective Disorders and Schizophrenia – Present and Lifetime version (K-SADS-PL)* is a standardized diagnostic interview for the assessment of psychiatric disorders in children and adolescents according to DSM-IV criteria [1]. Symptoms can be classified as “not present”, “possible”, “in remissions” or “certain*”.* The instrument was used at baseline to confirm OCD diagnoses and to evaluate the presence of comorbidity. To this end, we only used the categories “certain*”* or “not present”. The K-SADS-PL has excellent inter-rater reliability [15, 25] and validity [21, 27].

### Statistical Analysis

We compared participants with missing and non-missing data based on baseline CY-BOCS total scores, gender, age, age of symptom onset, and comorbidity. These comparisons revealed no significant differences at baseline, nor between participants with and without missing data. Thus, we treated missing data as randomly missing.

We calculated the percentage of patients with clinical range OCD symptoms defined as CY-BOCS ≥ 16, and those in remission defined as CY-BOCS ≤ 12 [11, 30]. We also determined the percentage of treatment responders, defined as those with a ≥ 35% symptom reduction on the CY-BOCS and a CGI-I (Clinical Global Impression scale – Improvement) rating of 1 or 2 indicating “improved” or “very much improved”). The effect size (d) was calculated by dividing the mean difference in CY-BOCS scores pre- and post-online CBT by the standard deviation of this difference.

Linear mixed effects models (LME) were used to assess the change in CY-BOCS total scores. These models included all available data and are suited for handling missing data and correlated repeated measurements. Fixed effects in the model were time (baseline and post-treatment), treatment cohort (NordLOTS vs. online CBT), and their interaction, with random effects for the intercept. An unstructured covariance model was used for correlated observations.

Multivariate χ2 test were applied to binary outcomes (e.g., remission). Missing data were replaced using multiple imputation with a sequential regression multivariate algorithm. This model incorporated all baseline demographics and outcome measures. We generated 20 imputations, following guidelines. The SAS 9.4 proc mi was used for imputation, and Rubin’s rules were applied to combine results from the 20 analyses, using the SAS 9.4 proc MIANALYZE procedure. A combined F statistic was reported, indicating statistical significance for a p-value less than 0.05. We also conducted LME to evaluate treatment outcomes during the 3-, 6-month and 1-year follow-ups. The model included the random effects (intercept) and fixed effects of time. To analyse the decrease in symptoms during follow-up, we introduced fixed effects of post-online CBT variable, also a time-varying predictor, which marked the passage of time after the start of follow-up. LME models were fit using the PROC MIXED procedure within the SAS Statistical Software, version 9.4. All tests were two-tailed, and *p* < .05 was considered to indicate statistical significance.

## Results

### Patient Characteristics

At baseline, the mean CY-BOCS total score was 24.6 (SD = 5.1) for NordLOTS and 26.1 (SD = 4.7) for online CBT (Table 2). We identified no statistically significant differences between the two samples, except for the presence of comorbid conditions. In the NordLOTS sample 63% had no comorbid diagnosis, versus only 34% in the online sample (*p* < .001). Accordingly, comorbid ADHD (*p* = .023) and ASD (*p* < .001) was more frequent in the online CBT sample. Three online CBT patients were receiving concurrent medication for non-OCD mental disorders, specifically aripiprazole 7.5 mg/d for TS, lisdexamphetamine 40 mg/d for ADHD and risperidone 0.5 mg/d for ASD). None of the patients received any specific medication for OCD.

**Table 2:** Comparison av patient characteristics of NordLOTS versus Online CBT

Variable	NordLOTS	Online CBT	*p*-value
n	269	29	
Age, year, mean (SD)	12.8 (2.7)	12.7 (2.61)	0.809
Female participants, n (%)	138 (51.3)	18 (62.1)	0.270
Male participants, n (%)	131 (48.7)	11 (37.9)	
CY-BOCS, mean (SD)			
Total score at baseline	24.6 (5.1)	26.1 (4.7)	0.141
Obsession score at baseline	12.3 (2.8)	13.1 (2.2)	0.091
Compulsion score at baseline	12.3 (2.7)	13.0 (2.7)	0.168
Comorbid disorders at baseline (K-SADS PL)			
Any anxiety disorder, n (%)	52 (19.3)	5 (17.2)	0.772
Any depressive disorder, n (%)	10 (3.7)	1 (3.4)	0.936
ADHD, n (%)	24 (8.9)	6 (20.7)	**0.023**
ODD/CD, n (%)	10 (3.7)	0	
Tic disorder, n (%)	49 (18.2)	7 (24.1)	0.438
ASD, n (%)	1 (0.4)	4 (13.8)	**< 0.001**
PTSD, n (%)	1 (0.4)	0	
Number of co-occurring diagnoses, n (%)			
None	163 (62.8)	10 (34.5)	**< 0.001**
1	62 (23.0)	14 (48.3)	
2	25 (9.3)	4 (13.8)	
≥3	13 (4.9)	1 (3.4)	

*Note* Significant differences (*p* < .05) are indicated with boldface

CY-BOCS = Children’s Yale-Brown Obsessive-Compulsive Scale; K-SADS-PL = Kiddie Schedule for Affective Disorders and Schizophrenia – Present and Lifetime Version; ADHD = Attention deficit/hyperactivity disorder; ODD/CD = Oppositional defiant disorder/Conduct disorder; ASD = Autism spectrum disorder; PTSD = Post traumatic stress disorder

### Acceptability

Of the 26 participants who completed the treatment, 23 children and 23 parents (88.5%) filled in the Client Satisfaction Questionnaire 8 (CSQ-8). Client satisfaction scores were high; ranging from 8 to 32, the mean total score was 29.47 (SD 3.01) for children and 30.13 (SD 2.38) for parents (Table 3).

**Table 3:** Client satisfaction questionnaire (CSQ-8): children’s and parents’ rating

Item	Children (*n* = 23) mean (SD)	Parents (*n* = 23) mean (SD)
1. Quality of service	3.52 (0.51)	3.70 (0.47)
2. Kind of service wanted	3.74 (0.45)	3.74 (0.45)
3. Needs met	3.57 (0.66)	3.61 (0.66)
4. Would recommend to friend	3.74 (0.45)	3.87 (0.34)
5. Satisfaction with help received	3.61 (0.58)	3.78 (0.42)
6. Dealt with problems	3.83 (0.39)	3.83 (0.39)
7. Overall satisfaction	3.78 (0.52)	3.83 (0.39)
8. Would return to program	3.70 (0.47)	3.78 (0.42)
Total score per patient (range 8–32)	29.47 (3.01)	30.13 (2.38)
Total score per item (range 1–4)	3.69 (0.11)	3.77 (0.08)

### Feasibility

Three participants dropped out, while 26 (90%) completed the treatment. One participant with ASD was not interested in treatment, but parents were, and he was included in the hope that he might change his mind. However, the goal to motivate him for exposure was never achieved and he dropped out of the study. The second dropout had a diagnosis of PTSD at baseline and was not able to adjust to the structure and requirements of the treatment. Most of the scheduled sessions were unattended, either forgotten or cancelled. Subsequent assessment concluded with a diagnosis of severe ADHD, ASD and family problems. The third drop out had no comorbidities diagnosed initially but was not able to engage in exposure work. Subsequently she was diagnosed with ASD. One of the 26 participants was deemed to be non-responder. He was diagnosed with ASD and Tourette’s syndrome prior to referral and had extremely incapacitating OCD symptoms: He barely was able to leave his room and needed parents’ assistance with basic hygiene routines. He had very slow progression and he was taken out of the study and started with medication after 22 sessions of online CBT.

**Fig. 2:**
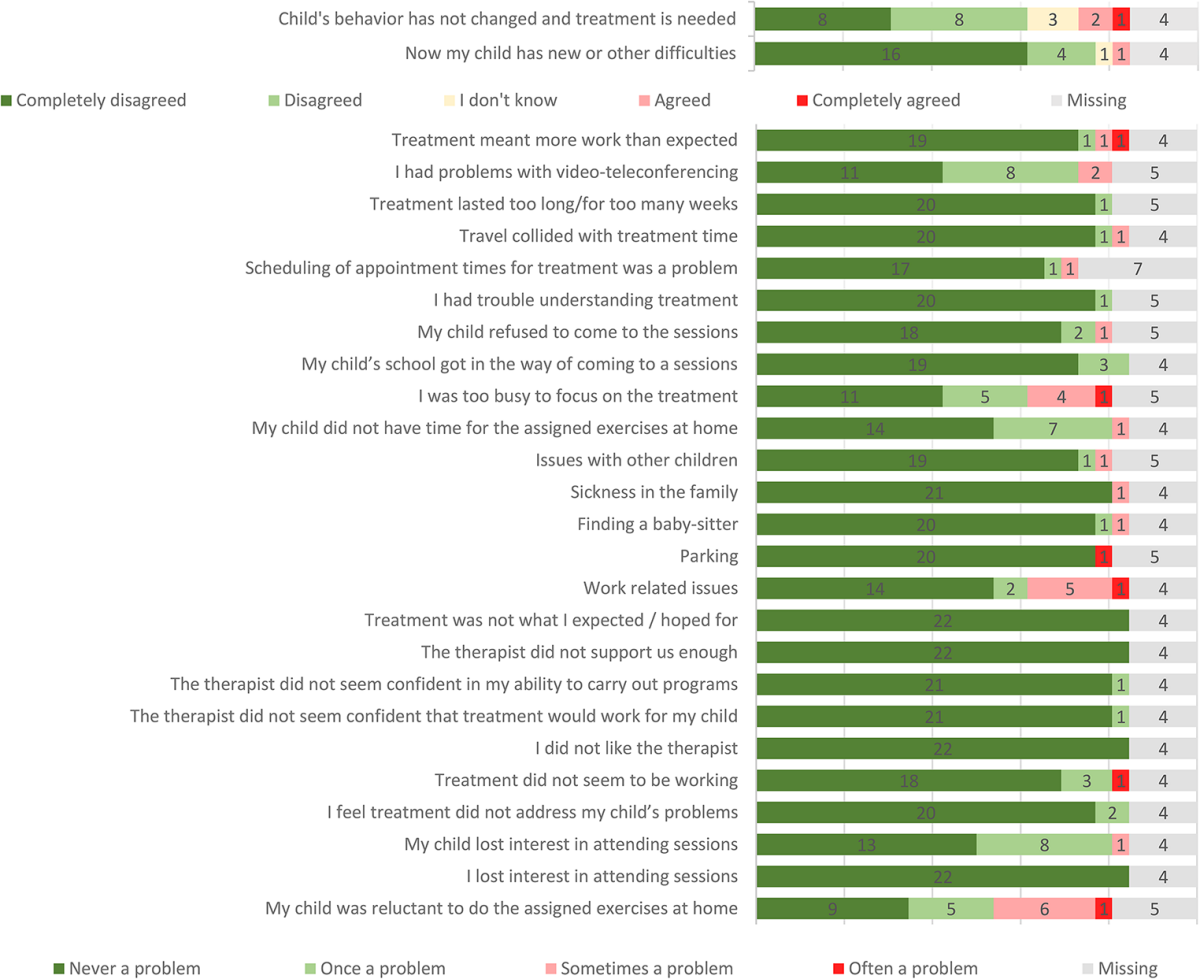
Modified Barriers to Treatment Participation Scale. Parents (*n* = 26)

The modified Barriers to Treatment Participation Scale showed that parents identified very few barriers for treatment participation, see Fig. 2.

**Fig. 3:**
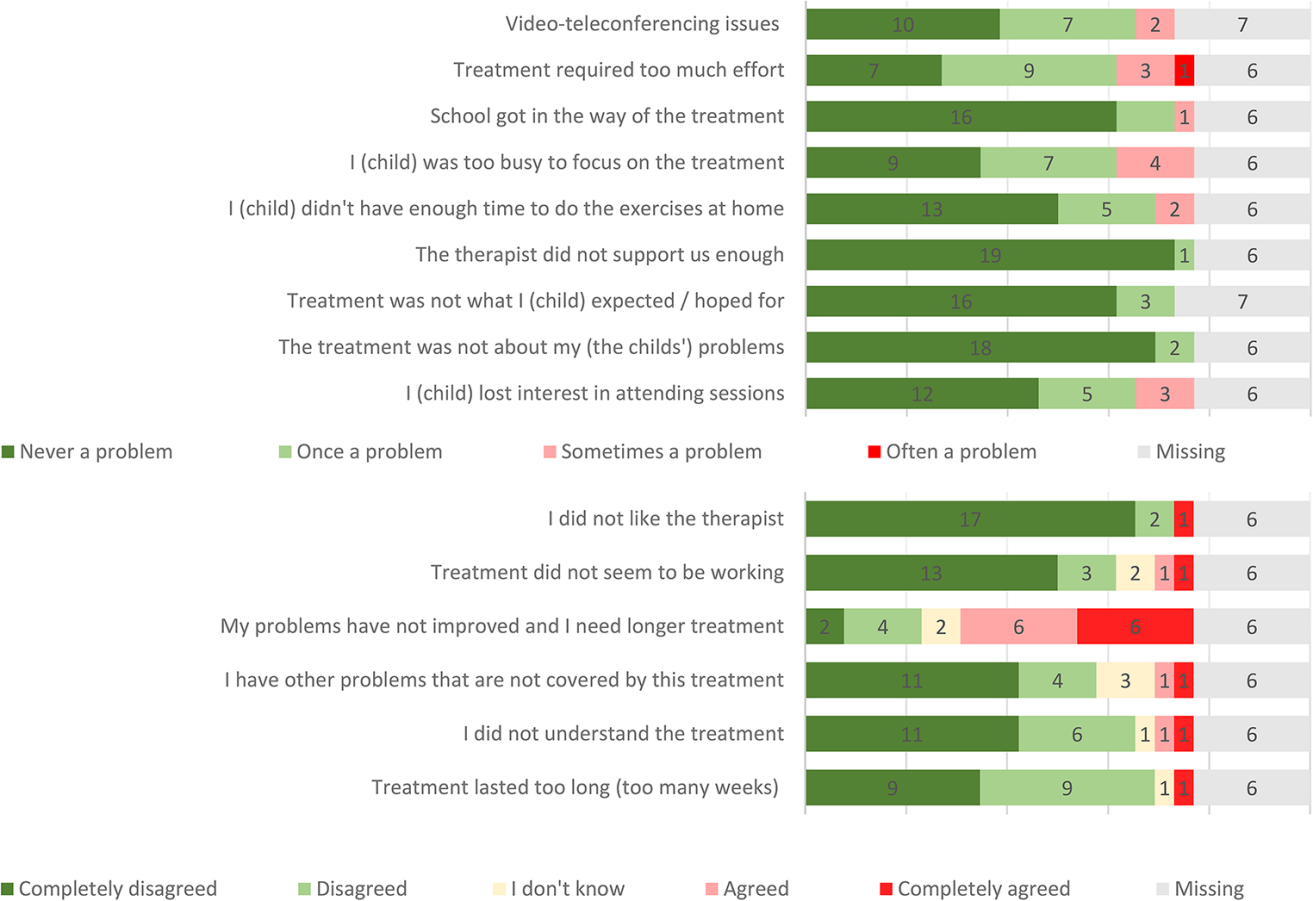
Modified Barriers to Treatment Participation Scale: Children (*n* = 26)

Also, the children identified few barriers for treatment participation, with one exception: the most frequent endorsed barrier to treatment participation was “My problems have not improved, and I need longer treatment”, endorsed by 12 children (Fig. 3).

### Effectiveness

**Fig. 4:**
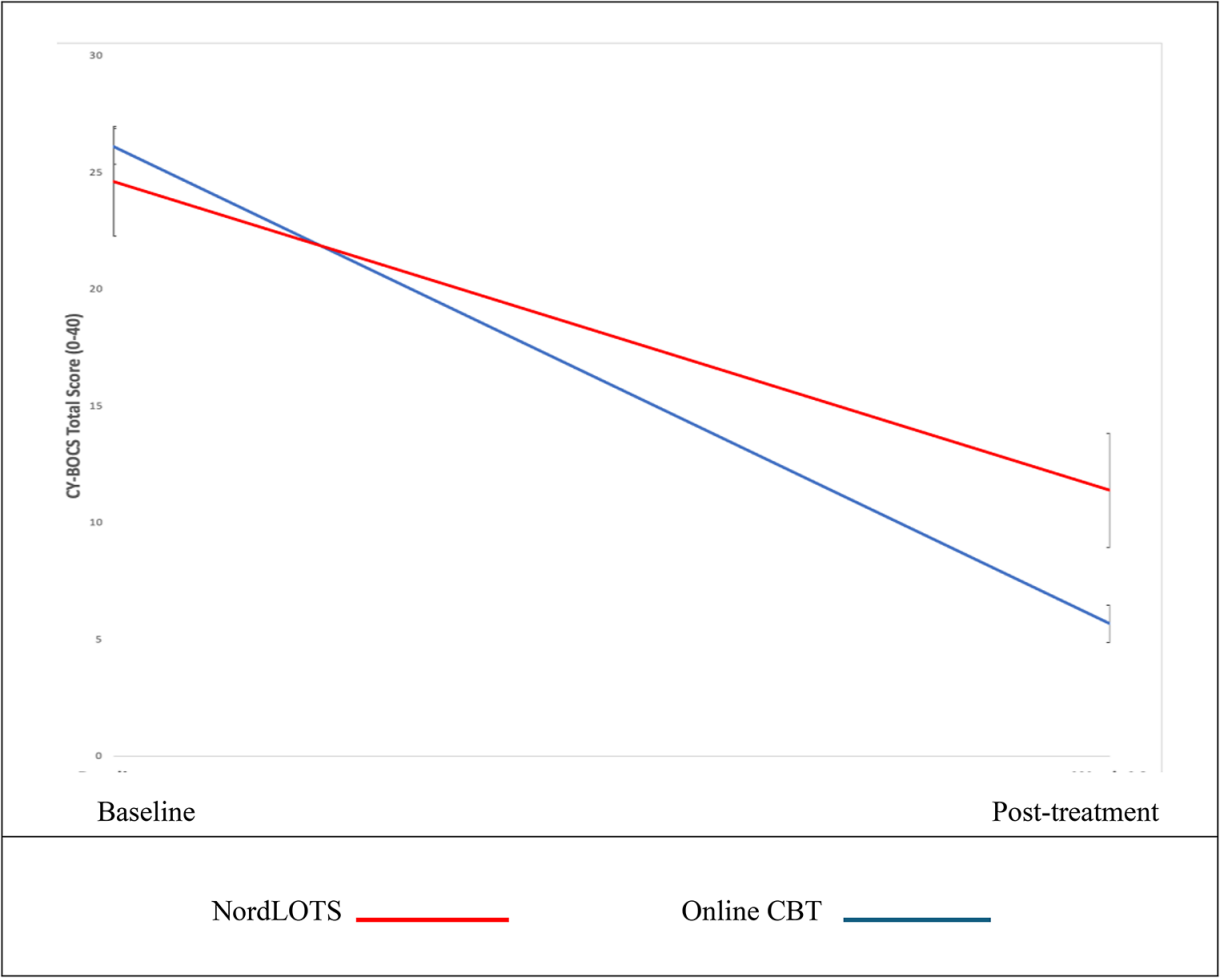
Adjusted intent-to-treat CY-BOCS total score across treatment cohorts

Adjusted intent-to-treat CY-BOCS total scores across treatment cohorts are shown in Fig. 4. Pairwise comparisons post-treatment showed that the difference between online CBT and NordLOTS treatment outcomes was 5.7 (95% CI 3.1, 8.3, *p* < .001). Further information on parameter estimates can be found in Table 4.

**Table 4:** Parameter estimates from fitting elevation and slope to the CY-BOCS

Parameter	Final model
**Composite model, estimate (SE)**	
Intercept	24.6245 (0.3866)***
Weeks	-0.9452 (0.03613)***
Group	1.4826 (1.2590)
Week*group	-0.5143 (0.1168)***
**Variance components**	
Level-1 – within person	32.3353 (2.7442)***
Level-2 – in initial status	7.8655 (2.4441)***

****p < .001

The online CBT group had a significantly higher response rate (90.0%) compared to the NordLOTS cohort (60.0%) (*p* = .002). We also examined differences in attrition and remission rates. Attrition rates were similar, with no significant differences between groups [c^2^ (1, 296) = 0.493, *p* = .483)]. The remission rate was statistically significantly higher (*p* = .231) for the online CBT group (80.9%) compared to the NordLOTS group (52.9%). We also examined if there were differences in the number of participants with symptoms still within clinical range (CY-BOCS total score 16 or above) after treatment. In the online CBT group 10.5% of participants were still within the clinical range, compared with 28.7% in the NordLOTS group. This difference was significant (*p* = .042).

**Table 5:** Post-treatment group-specific outcomes and response rates

Primary outcomes	Online CBT	NordLOTS	Effect sizes (95% CD) ^b c^
	Estimated mean or rate (95% CI)^a^		
CY-BOCS total score	5.67 (3.23, 8.12)	11.39 (10.59, 12.19)	-0.86 (-0.45, -1.28)
Reduction from baseline CY-BOCS total score	-20.43 (-17.37, -23.49)	-13.23 (-12.24, -14.23)	-0.87 (-0.48, -1.26)
Symptoms in clinical range (CY-BOCS ≥ 16)	10.5% (0%, 21.7%)	28.7% (23.3%, 34.1%)	-0.47 (-0.35, -0.59)
Remission (CY-BOCS ≤ 12)	80.9% (66.5%, 95.2%)	57.9% (52.0%, 63.8%)	0.51 (0.35, 0.66)
Response rate	90.0% (79.1%, 100.0%)	60.0% (54.1%, 65.9%)	0.73 (0.60, 0.85)
Attrition rate	10.3% (*n* = 3)	9.7% (*n* = 26)	

a For CY-BOCS total score, estimated mean score at post-treatment from the fitted LMM. For the categorical outcomes, the estimated rate at post-treatment

b For CY-BOCS total score, between-groups difference in estimated mean score at post-treatment. For the categorical outcomes, between-groups difference in rate at post-treatment

c Positive effect size suggests that eCBT was more effective

The CY-BOCS mean estimate either remained stable or decreased during follow-up, with values of 5.17 (95% CI 3.65, 6.69) at 3 months, 4.36 (95% CI 2.90, 5.82) at 6 months, and 2.85 (95% CI 0.94, 4.77) at 12-months, respectively. All participants retained their responder status throughout the 12-month follow-up period: Participants either maintained or showed improvement in their CY-BOCS scores during the follow-up period, except for two children whose symptoms worsened from the post-treatment assessment to the 12-month follow up. However, in both children the deterioration was limited, and they maintained a subclinical symptom level with CY-BOCS total scores of 11 and 12 respectively. A synopsis of the participants characteristics, number of treatment sessions and outcome in terms of CY-BOCS scores post treatment and during follow up is given in Table 1.

## Discussion

The main goal of this study was to explore acceptability, feasibility, and potential effectiveness of online CBT. As a first step in examining the effectiveness of this new treatment delivery package, we compared its outcomes to well-established face-to-face CBT, i.e., NordLOTS. Online CBT and NordLOTS samples were comparable in terms of OCD severity scores at baseline and gender ratio but were significantly different in the overall rate of comorbidity. In addition, in the online CBT study participants with comorbid ASD were accepted, while this was an exclusion criterion in the NordLOTS sample. However, a diagnosis of Pervasive Development Disorder – Not Otherwise Specified (PDD-NOS) was allowed in the NordLOTS if symptoms of OCD were most impairing. Therefore, there was one patient with comorbid ASD in the NordLOTS sample. Of the 29 participants included in the online CBT group, four had a diagnosis of ASD at baseline. In addition, comorbid ADHD was more frequent in the online CBT sample yielding a higher total number of co-occurring diagnoses in the online CBT group than in the NordLOTS sample.

### Acceptability

Due to the COVID-19 lockdowns in Norway in the first part of the study period, participants and their parents had no option for another treatment modality than online treatment, except the choice to wait until the lockdown would be revoked. We do not know whether the limited choice influenced the acceptability rating. Still, the client satisfaction total scores in the CSQ-8 were close to the upper range of 32, with mean score 29.47 for children and 30.13 for parents (Table 3), demonstrating a very high acceptability of the online intervention. A fact that might have contributed to this high acceptance was the surprisingly low frequency of technical problems, probably an impact of the fast-growing expertise with internet-based communication in the general population during the COVID pandemic.

### Feasibility

Dropout rates from treatment did not differ significantly between online CBT and NordLOTS (10.3% versus 9.7% respectively), indicating that online CBT was feasible for most participants, comparable to standard CBT. The three participants who dropped out were subsequently diagnosed with ASD. Outcomes of CBT for children and adolescents with OCD and comorbid ASD have been associated with lower response rates [33]. The conclusion of a review examining the effectiveness of CBT for individuals with ASD and comorbid OCD was that CBT needs to be modified and adjusted to the needs of children with ASD, for example, with regard to session structure, visual aids and increased parental involvement [24]. All three dropouts had passed the K-SADS interview unnoticed. Probably it could have made a difference if the diagnosis had been established before the referral for OCD treatment, allowing for a better adjustment of the CBT to these children. At least, of the three participants with an ASD diagnosis established at baseline, two responded very well to treatment (CY-BOCS total score reduction from 30 to 8 and from 19 to 10, respectively), and both maintained and improved treatment gains at the 12 month follow up. The third participant with ASD and incapacitating OCD showed at least some improvement (CY-BOCS total score from 35 to 24), before he was removed from the study because he had reached the maximum limit of sessions and initiated medication.

Parents rated the treatment highly feasible, according to the modified Barriers to Treatment Participation Scale. Parents identified only a few barriers for treatment participation. Of the three most frequently endorsed barriers, two were not linked directly to the treatment, but to parents’ individual circumstances as “I was too busy to focus on the treatment” and “work related issues”. The third frequently endorsed barrier, “My child was reluctant to do the assigned exercises at home” is a problem closely related to the demand for challenging exercises and CBT aims to overcome this reluctance during the treatment course. Surprisingly, almost half of the children (*n* = 12) endorsed the item “My problems have not improved, and I need longer treatment”, despite the favorable outcome in terms of CY-BOCS reduction in most cases. This seems to reflect that typically children need to practice their newly learned skills over time and implement the treatment gains in their everyday life, before they really feel they have overcome OCD.

A quarter of patients who were offered online CBT declined, preferring to wait for face-to-face treatment. We don’t know exactly what influenced these decisions. However, comorbidity could have played a role. Two of the nine who preferred to wait, had an ASD diagnosis. In our experience, children with ASD frequently are more reluctant in seeking treatment and need more time and effort for motivation. Another point could be timing. The number of those who wished to wait was higher (*n* = 7) in the last inclusion period for the online study, when COVID-19 lockdown measures were mitigated, resulting in short waiting time for face-to-face or combined treatment.

### Effectiveness

In the online CBT cohort 90% of the participants responded to treatment and an estimated mean of 80.9% were in remission (CY-BOCS ≤ 12) at post treatment evaluation (Table 5). This was an unexpectedly high number compared to NordLOTS, our bench-marking study, where the response rate was 60%.

Also, when comparing to other studies using Internet technology to deliver CBT for children with OCD, online CBT had a favorable outcome: Storch et al. [39] reported a 56.1% reduction of CY-BOCS total scores after 14 sessions of online CBT. Farrell et al. [12] reported a 49% reduction of CY-BOCS scores after 3 face-to-face CBT sessions, followed by a 3-week maintenance program online. The corresponding figures were 78.3% reduction of CY-BOCS scores from baseline to post-treatment evaluation for online CBT and 53.7% for NordLOTS. Hollmann et al., [17] reported remission rates (CY-BOCS total score of 12 or less) of 68% in the treatment group and 79% in the subsequently treated waiting list group after 14 sessions of therapist-delivered online CBT. Aspvall et al. [2] randomized 152 children and adolescents with OCD to either online CBT followed by traditional face-to face CBT if necessary (stepped-care group) or 16 weeks face-to face CBT alone. At 3-month follow-up, 34 participants (46%) in the stepped-care group and 23 (30%) in the face-to face CBT group were non-responders. Non-responders in both groups were offered an additional course of face-to-face treatment. At 6 months follow up, the mean CY-BOCS score was 11.57 in the stepped-care group versus 10.57 in the face-to face CBT group, a difference that was noninferior to face-to face CBT alone. Other studies applying various degrees of Internet technology to deliver CBT to children with OCD reported somewhat lower reductions in CY-BOCS scores after treatment [6].

We can only hypothesize why online CBT participants achieved such a remarkable remission rate. Several factors may have contributed to this outcome. First, almost a quarter of the invited participants preferred to wait until face-to-face treatment would be available again after lockdown. Thus, the most motivated patients may have participated, constituting a potential selection bias in our sample. However, in terms of severity and presence of comorbidity at baseline, the sample did not differ from the much larger sample of NordLOTS we used as reference sample for benchmarking. Based on feed-back from the young people as well as their parents, even those who were skeptical of online treatment sessions, became gradually more confident with this intervention, enjoying the reduced logistical demands compared to in person sessions. Parents miss significant work to attend in person sessions, while online sessions offered convenient flexibility, especially for families living in more distant places. Besides reducing geographical barriers, online treatment had an enormous advantage facilitating exposure directly modelled and supervised by the therapist at home, i.e. in a naturalistic environment where OCD symptoms usually are most prevalent. Practicing and implementing exposure in daily life is essential for a successful treatment. Online support from the therapist when practicing exposure at home, may have the potential to contribute to the favorable outcome we have reported and to reduce treatment failure and dropouts.

The COVID-19 pandemic has catalyzed digital transformation in all aspects of our society, including CAMHS. The pandemic highlighted the need for treatment without direct contact. Delivering online treatment offers not only safe treatment, but also more convenient and accessible treatment, as well as more possibilities for therapist-assisted exposure exercises in the patient’s home environment. Our initial concerns, that it would be more difficult to establish the necessary motivation and therapeutic relation for a demanding treatment without direct face-to-face contact, potentially resulting in less favorable outcomes, proved to be unfounded. Communication and establishing rapport via online sessions may be somewhat different, but not impossible. Online treatment providers should use the advantages offered by online communication: The therapist can for example meet the family in their safe home environment. Learning the strengths and interest of the young people can actually be facilitated by the digital environment. The participants can, for example, show cuddly toys, pets, drawings or other attachment items on the screen that may contribute to the establishment of the therapeutic relation. However, therapists should be aware that online treatment does not work for all young people. While it may lower the threshold to engage in treatment for most young people, some may find it impossible to appear on a screen, especially in the context of anxiety or conduct disorders.

### Strengths and Limitations

A strength of this study is that assessment methods and applied instruments were almost the same in both samples, with an independent evaluation of the main outcome (CY-BOCS score). Since the treatment concept of online CBT is derived from NordLOTS CBT and a similar Dutch treatment protocol, treatment strategies for both studies were similar, except delivery mode. Also, sample characteristics were similar, irrespective of sample size, facilitating comparison between samples. OCD severity was comparable. That the online sample included more comorbid disorders is consistent with an at least not easier to treat sample.

The main limitation of our study is the small online CBT sample size and a lacking direct comparison group. However, as described in the introduction, these limitations derive from the concept of the online CBT study as a pilot feasibility trial motivated by the need of a sudden change of delivering treatment during the COVID pandemic. Still, it gives the opportunity to explore preliminary effectiveness, with comparison to a benchmark outcome before going on with much more resource demanding randomized and controlled studies. NordLOTS has documented that the treatment gains were durable over a follow-up period of 3 years [32]. While we compare acute outcomes of online CBT measured directly post-treatment with NordLOTS outcomes, we have also documented, that treatment gains remained stable during follow-up over 12 months.

## Summary

In a pilot open trial benchmarked against NordLOTS, online CBT has shown superiority compared to well-established standard in person CBT. Contrary to our hypothesis, we found significantly higher mean reduction of CY-BOCS scores in the online CBT group. Altogether, our pilot data suggest that it is possible to establish rapport and the trusting therapeutic relationship necessary for demanding exposure-based treatment by completely online contact. Online CBT seems to have the potential to be at least as effective in reducing OCD symptoms compared to standard CBT. However, whether this result is due to low sample size or will stand, needs to be examined in randomized head-to-head comparisons between larger groups.

## Data Availability

The data and materials are available from the corresponding author.
